# Lag effect of ambient temperature on respiratory emergency department visits in Beijing: a time series and pooled analysis

**DOI:** 10.1186/s12889-024-18839-6

**Published:** 2024-05-21

**Authors:** Xuan Li, Yongming Zhang, Zhenbiao Tian, Jianping Wang, Jinhua Zhao, Yuanjun Lyu, Ying Ni, Yuming Guo, Zhuang Cui, Wenyi Zhang, Changping Li

**Affiliations:** 1 Department of Epidemiology and Biostatistics, School of Public Health, Tianjin Medical University, Heping District, Tianjin, 300070 P.R. China; 2 Department of Pulmonary and Critical Care Medicine, Center of Respiratory Medicine, China-Japan Friendship Hospital, National Clinical Research Center for Respiratory Diseases, Beijing, China; 3 Beijing Red Cross Emergency Center, Beijing, 100085 China; 4 Department of Endocrinology, Tianjin Hospital, Tianjin, China; 5 Department of Epidemiology and Preventive Medicine, School of Public Health and Preventive Medicine, Monash University, Melbourne, Australia; 6 Chinese PLA Center for Disease Control and Prevention, 20 Dong-Da Street, Fengtai District, Beijing, 100071 People’s Republic of China

**Keywords:** Respiratory diseases, Emergency department visits, Ambient temperature, Meteorological factors, Distributed lag non-linear model, Meta-analysis

## Abstract

**Background:**

Although the association between ambient temperature and mortality of respiratory diseases was numerously documented, the association between various ambient temperature levels and respiratory emergency department (ED) visits has not been well studied. A recent investigation of the association between respiratory ED visits and various levels of ambient temperature was conducted in Beijing, China.

**Methods:**

Daily meteorological data, air pollution data, and respiratory ED visits data from 2017 to 2018 were collected in Beijing. The relationship between ambient temperature and respiratory ED visits was explored using a distributed lagged nonlinear model (DLNM). Then we performed subgroup analysis based on age and gender. Finally, meta-analysis was utilized to aggregate the total influence of ambient temperature on respiratory ED visits across China.

**Results:**

The single-day lag risk for extreme cold peaked at a relative risk (RR) of 1.048 [95% confidence interval (CI): 1.009, 1.088] at a lag of 21 days, with a long lag effect. As for the single-day lag risk for extreme hot, a short lag effect was shown at a lag of 7 days with an RR of 1.076 (95% CI: 1.038, 1.114). The cumulative lagged effects of both hot and cold effects peaked at lag 0–21 days, with a cumulative risk of the onset of 3.690 (95% CI: 2.133, 6.382) and 1.641 (95% CI: 1.284, 2.098), respectively, with stronger impact on the hot. Additionally, the elderly were more sensitive to ambient temperature. The males were more susceptible to hot weather than the females. A longer cold temperature lag effect was found in females. Compared with the meta-analysis, a pooled effect of ambient temperature was consistent in general. In the subgroup analysis, a significant difference was found by gender.

**Conclusions:**

Temperature level, age-specific, and gender-specific effects between ambient temperature and the number of ED visits provide information on early warning measures for the prevention and control of respiratory diseases.

**Supplementary Information:**

The online version contains supplementary material available at 10.1186/s12889-024-18839-6.

## Highlights

1. Cold weather had a longer lag time and hot weather had a shorter lag time but a stronger effect.

2. Extreme temperatures had a greater effect on emergency room visits for respiratory disease than moderate temperatures.

3. The elderly population were more sensitive to cold and hot weather.

**Supplementary Information:**

The online version contains supplementary material available at 10.1186/s12889-024-18839-6.

## Introduction

The prevalence and disease burden of respiratory diseases is high [1], with approximately 3.9 million deaths from respiratory diseases each year, ranking third in mortality among non-communicable diseases, after cardiovascular diseases and cancer [2]. Although the incidence of respiratory diseases has declined over the past two decades, respiratory diseases remain one of the most prevalent diseases in developing countries [3]. In recent years, with the frequent occurrence of global extreme weather phenomena, climate change has been regarded as one of the most serious public health problems of the twenty-first century [4]. The consequences of climatic conditions on human health are now a study priority. Much research on the impact of meteorological conditions on health have been carried out globally [5–7]. Global warming has caused the average global temperature to increase every year, and since the changes in ambient temperature are expected to be the most direct way in which climate change directly affects human health, the focus has also been placed on how ambient temperature affects human health [8].

Although the human body can adapt to ambient temperature changes, the respiratory system, due to direct or indirect interactions with the external atmospheric environment, can promote or exacerbate respiratory disease or increase the risk of exposure to respiratory disease risk factors [9], and then increase the incidence of respiratory diseases [10]. However, the available epidemiological evidence is inconsistent. An Iranian study found a strong negative correlation between maximum temperature and respiratory mortality and a positive correlation between minimum temperature and mortality [11]. Research from northwest China discovered that both cold and hot weather increased the chance of hospitalization for respiratory disorders, with cold weather having more adverse effects and lasting longer than hot weather [12]. Another study conducted in Beijing discovered that a rise in temperature was closely associated with an increase in the risk of respiratory death [13]. Furthermore, although several studies explored how extreme temperature affected the prevalence of respiratory diseases [14–16], less focus has been placed on the likelihood of developing respiratory diseases linked with certain temperature levels (e.g., extreme cold, moderate cold, moderate hot, and extreme hot). Consequently, it is necessary to elucidate the connection between the risk of respiratory diseases and the various levels of ambient temperature.

The annual mean temperature increased gradually at a pace of 0.39 °C/10 annual in Beijing from 1960 to 2008, which is higher than the rate of global warming (0.13 °C/10 annual) [17, 18]. Therefore, it is important and essential to determine how the ambient temperature affects respiratory diseases in Beijing [18]. The association between ambient temperature and hospitalization and death from respiratory diseases has been the subject of several studies around the world [4, 11, 19]. However, morbidity risk can describe a broader range of health events than mortality, which can only describe the most severe health outcomes [20]. In addition, fewer studies have been conducted on respiratory emergency department (ED) visits. Taking into account the features of the healthcare system, ED visits are more sensitive to respond to the impact of environmental factors on human health [21]. To our knowledge, only 2 studies had explored the influence of extreme temperatures on ED visits for respiratory diseases in Beijing [18, 22], and the studies were conducted before 2012 with inconsistent findings.

Previous studies have found that the influence of ambient temperature on the risk of respiratory diseases is nonlinear and lagged [23–25], and the other meteorological factors and atmospheric pollutants are also likely to be correlated with the risk of respiratory diseases [26–28]. Therefore, a distributed lag non-linear model (DLNM) was used to explore the effect of the different levels of ambient temperature on the risk of respiratory diseases from the largest emergency facility in Beijing during 2017–2018. Furthermore, subgroup analyses were carried up by sex and age. Finally, a meta-analysis was used to summarize the results of other similar studies in China. This study will serve as a scientific foundation for establishing and improving relevant preventive interventions in Beijing.

## Materials and methods

### Data collection

Daily respiratory ED visits were collected from the Beijing Red Cross Emergency Medical Center from January 1st 2017 to December 31st 2018. The center has more than 100 emergency stations throughout Beijing. Date of appointment, type of respiratory illness, patient gender, and age were included in the above dataset. The illnesses were filtered in accordance with the 10th International Classification of Diseases, respiratory diseases (ICD-10: J00-J99). These include acute upper respiratory infections (J00-J06), influenza and pneumonia (J09-J18), Other diseases of upper respiratory tract (J30-J39), chronic lower respiratory diseases (J40-J47), lung diseases due to external agents (J60-J70), other respiratory diseases principally affecting the interstitium (J80-J84), suppurative and necrotic conditions of lower respiratory tract (J85-J86), other diseases of pleura (J90-J94), other diseases of the respiratory system (J95-J99).

Daily meteorological data for this study was collected from the National Meteorological Information Center of China [29]. Daily air pollution data was collected from the China National Real-Time Urban Air Quality Release Platform [30]. The meteorological data included the daily averages for temperature, relative humidity (RH), sunshine duration (SD), air pressure, precipitation, and wind speed. The daily average values of meteorological factors are calculated based on the average values of meteorological stations in 16 districts of Beijing. In addition, the air pollution dataset includes daily averages of nitrogen dioxide (NO2), sulfur dioxide (SO2), ozone (O3), carbon monoxide (CO), PM2.5, and PM10, which are based on the average of the air quality values from all 35 air pollution stations in Beijing. The meteorological data and pollution data in this article are both from the official website, covering the entire year from January 1, 2017 to December 31, 2018. Therefore, there is no missing data in this article. A map of the main emergency stations and all environmental monitoring stations is shown in Supplementary Figure S1.

### Statistical analysis

First, the collected respiratory ED visits and meteorological data were cleaned and put into daily-scale time series data. Demographic, meteorological, and air pollution statistics were all subjected to descriptive statistical analysis. A possible correlation was revealed between daily mean temperature and daily respiratory ED visits through time series graphs and scatter plots. The Spearman test was used to investigate the relationship between the factors. The components with Spearman correlation values > 0.6 were disregarded from the model [31] to prevent multicollinearity between various variables [27].

Second, considering the relationship between temperature and respiratory diseases has both nonlinear and lagged effects, the DLNM model was used to quantify the effect of cold and hot weather on respiratory ED visits in Beijing from 2017 to 2018. The model was fitted by a quasi-Poisson time-series generalized additive model, which included a crossbasis function to explore the lagged effects and a crosspred function to make predictions. Combined with the Spearman results, the mean relative humidity, daily precipitation, sunshine duration, PM_2.5_, and SO_2_ were finally determined to be included in the model. The maximum lag days were fixed at 21, and this number was chosen in light of evidence from earlier research that cold weather has a delayed impact lasting for several weeks [32–34]. These analyses were further stratified by sex and age. And Z-test was used to compare the differences within subgroups. In addition, adjustments were done to account for the day of the week and holiday characteristics. According to the idea of minimized generalized cross-validation (GCV) fraction, a natural cubic spline with 4 degrees of freedom in temperature space and 3 degrees of freedom in lag space was used to simulate the nonlinear and lagged effects on the ambient temperature on day t. The reference temperature was set to the daily mean temperature value of 1 ℃ corresponding to the lowest relative risk (RR) of ED visits for respiratory diseases. The basic model is as follows.

$$\begin{array}{c} \log [E (Y_{t})] = \alpha + \beta\ \ast\ \text{Temp}_{t,l} + ns(RH,3) + ns(PRE,3) + ns(SD,3)\\ + ns(PM_{2.5},3) + ns(SO_{2},3)\\ +ns(\text{Time}\ , 4^{\ast}2) + \gamma\,\text{DOW}_{t} + \delta\,\text{Holiday}_{t} \end{array}$$

Where Y is the daily count of respiratory ED visits on day t, and t is the observation day. Y_t_ is assumed to obey a quasi-Poisson distribution each day, α is the intercept, and Temp_t,l_ is the temperature cross basis matrix in the DLNM model, where l is the number of the lag days. Ns is the natural cubic spline. Set the degree of freedom (df) for relative humidity, daily precipitation, sunshine duration, PM_2.5_, and SO_2_ as 3. According to the principle of minimum sum of absolute values of the partial autocorrelation function (PACF), the df of time is chosen as 4 times per year to control for long-term trends and seasonality (Supplementary Figure S5); DOW is the day of the week, with Sunday as the reference; Holiday is a binary variable, where "1" denotes the legal holiday day; β, γ, δ are the coefficients.

To investigate the model's robustness, we performed several sensitivity analyses. setting the reference temperature to the ambient temperature associated with the lowest risk of morbidity. First, the df was adjusted from 2 to 6 for the exposure and lag dimensions, respectively. Second, three types of corrections for interaction terms are performed in the DLNM model. These are the DLNM model corrected for the interaction between temperature and PM_2.5_, the DLNM model corrected for the interaction between temperature and SO_2_. and the model corrected for the interaction terms between temperature and PM_2.5_ and temperature and SO_2_. Third, set the maximum lag days to 10 days.

Finally, a meta-analysis of all studies on the relationship between respiratory ED visits and daily mean temperature in China was performed to obtain an estimate of the overall effect of ambient temperature on respiratory ED visits in China and to further complement the results of this study. The following databases were used for the search: PubMed, Web of Science, CNKI, and Wanfang Data. The search time was not limited, and the search criteria were the relationship between daily mean temperature and respiratory ED visits in China. The search process is described in Supplementary Figure S2.

The aforementioned data analysis and models were performed by using the R software (version 4.1.2). All statistical tests were two-sided, and statistical significance was defined as *p* < 0.05.

## Results

### Descriptive analysis

Table 1 describes the demographic information on ED visits for respiratory diseases, meteorological information, and air pollution information throughout the research period. A total of 19,367 ED visits for respiratory diseases were reported in Beijing. The time series distribution and scatter plot of the number of visits versus daily temperature were shown in Supplementary Figure S3, S4.

**Table 1:** The number of daily visits and environmental variables for respiratory diseases and subgroups in Beijing from 2017 to 2018

Variables	Number/Days	Mean ± SD	Minimum	P25	Medium	P75	Maximum
Demographic information							
Total	19367	26.53 ± 9.20	5	20	26	31	69
Male	11314	15.50 ± 5.71	3	11	15	19	45
Female	8053	11.03 ± 4.90	1	7	10	14	30
Age < 65	3080	4.22 ± 2.38	0	3	4	6	14
Age ≥ 65	16287	22.31 ± 8.11	4	17	21	27	59
Meteorological factors							
Mean temperature (℃)	730	12.07 ± 11.81	-12	0.48	13.72	22.97	30.9
Relative humidity (%)	730	52.76 ± 19.47	14.67	36.33	50.67	70	94.67
Sunshine duration (h)	730	7.07 ± 3.50	0	4.58	7.83	9.66	13.23
Air pressure (hPa)	730	993.76 ± 9.45	974.07	985.47	994.47	1000.82	1019.13
Precipitation (mm)	730	1.61 ± 6.25	0	0	0	0	60.33
Wind speed (m/s)	730	1.70 ± 0.63	0.47	1.27	1.57	2	5.03
Air pollution factors							
NO2 (μg/m3)	730	40.63 ± 19.58	6	27	36	50	145
SO2 (μg/m3)	730	5.95 ± 6.45	1	2	4	7	81
O3 (μg/m3)	730	61.60 ± 37.77	3	33.25	55	84	181
CO (mg/m3)	730	0.87 ± 0.63	0.2	0.5	0.76	1.02	7.28
PM2.5 (μg/m3)	730	52.58 ± 49.00	3	20	40	67.75	430
PM10 (μg/m3)	730	80.92 ± 68.70	0	41	65.5	99.75	858

*SD* Standard deviation, *Px* xth percentile

Table 2 demonstrates the Spearman correlation between respiratory ED visits and meteorological and air pollution factors. The daily mean temperature was significantly and positively correlated with daily mean relative humidity, sunshine duration, daily precipitation, and O_3_, and significantly and negatively correlated with daily mean air pressure, daily mean wind speed, SO_2_, and NO_2_.

**Table 2:** Spearman correlation between respiratory ED visits, meteorological factors and the pollutants in Beijing

Variables	Respiratory ED visits	Mean temperature	Relative humidity	Sunshine duration	Air pressure	Wind speed	Precipitation	PM_2.5_	PM_10_	SO_2_	NO_2_	CO	O_3_
Respiratory ED visits	1.00												
Mean temperature	-0.28^***^	1.00											
Relative humidity	-0.20^***^	0.50^***^	1.00										
Sunshine duration	0.01	0.17^***^	-0.46^***^	1.00									
Air pressure	0.19^***^	-0.88^***^	-0.41^***^	-0.15^**^	1.00								
Wind speed	0.10^**^	-0.19^***^	-0.65^***^	0.28^***^	0.12^**^	1.00							
Precipitation	-0.16^***^	0.38^***^	0.54^***^	-0.39^***^	-0.36^***^	-0.16^***^	1.00						
PM_2.5_	-0.01	0.09	0.40^***^	-0.35^***^	-0.17^***^	-0.37^***^	0.05	1.00					
PM_10_	-0.01	0.06	0.04	-0.09^*^	-0.16^***^	-0.12^***^	-0.15^***^	0.78^***^	1.00				
SO_2_	0.21^***^	-0.45^***^	-0.27^***^	-0.12^**^	0.31^***^	0.02	-0.30^***^	0.46^***^	0.52^***^	1.00			
NO_2_	0.03	-0.25^***^	0.12^**^	-0.21^***^	0.17^***^	-0.44^***^	-0.22^***^	0.65^***^	0.61^***^	0.61^***^	1.00		
CO	0.08^*^	-0.05	0.45^***^	-0.48^***^	-0.02	-0.48^***^	0.07	0.85^***^	0.58^***^	0.55^***^	0.69^***^	1.00	
O_3_	-0.12^**^	0.74^***^	0.08^*^	0.35^***^	-0.70^***^	0.20^***^	0.21^***^	-0.03	0.04	-0.29^***^	-0.47^***^	-0.23^***^	1.00

Correlation coefficients greater than 0.6 represent covariance and are indicated in bold type

^*^p < 0.05

^**^p < 0.01

^***^p < 0.001

### Association between ambient temperature and daily respiratory diseases number

**Fig. 1:**
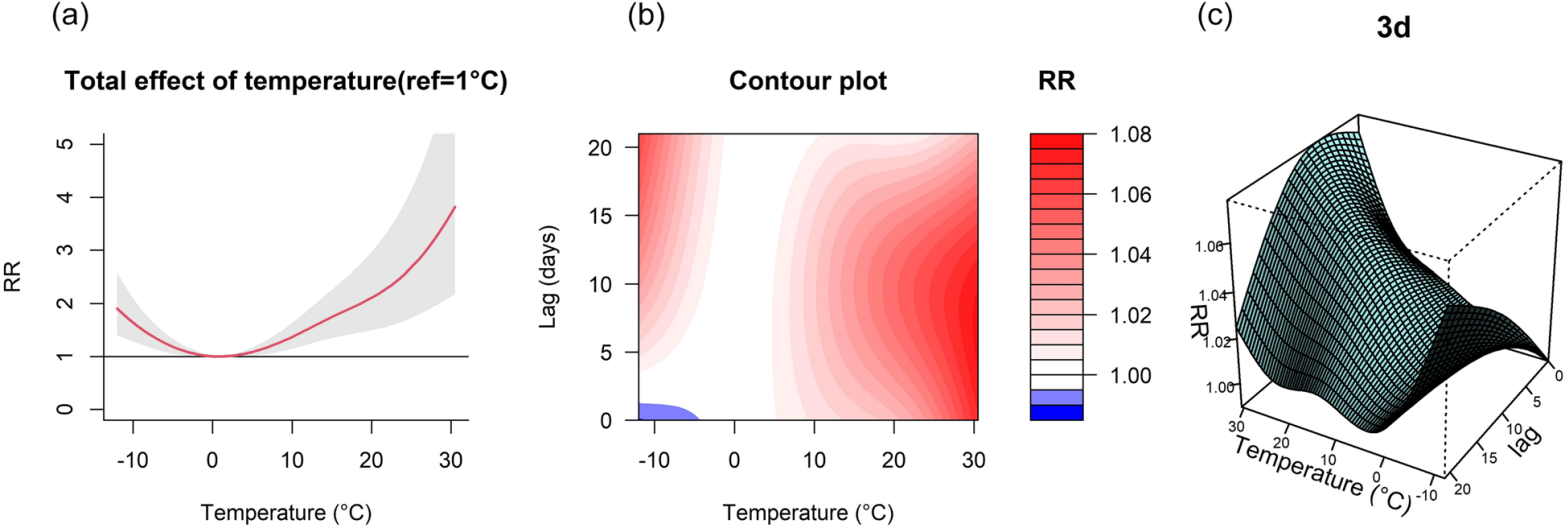
The delayed impact of ambient temperature on ED visits for respiratory diseases in Beijing

Figure 1a shows the exposure–response curve for the relative risk between the daily mean temperature and ED for respiratory diseases, with a J-shaped curve. The risk of disease onset was lowest at 1 °C (RR = 0), so 1 °C was set as the reference temperature. There were 8 days of extremely cold weather (temperatures below the 1st percentile) and 9 days of extremely hot weather (temperatures above the 99th percentile). The RR tends to increase in cold and hot weather. It is suggested that both cold and hot are risk factors for the development of respiratory diseases. Figure 1b and c show that both cold and hot temperatures have a lag effect on respiratory diseases, with cold temperatures having longer lag days than hot temperatures, but overall, the effect of hot temperatures is stronger.

**Fig. 2:**
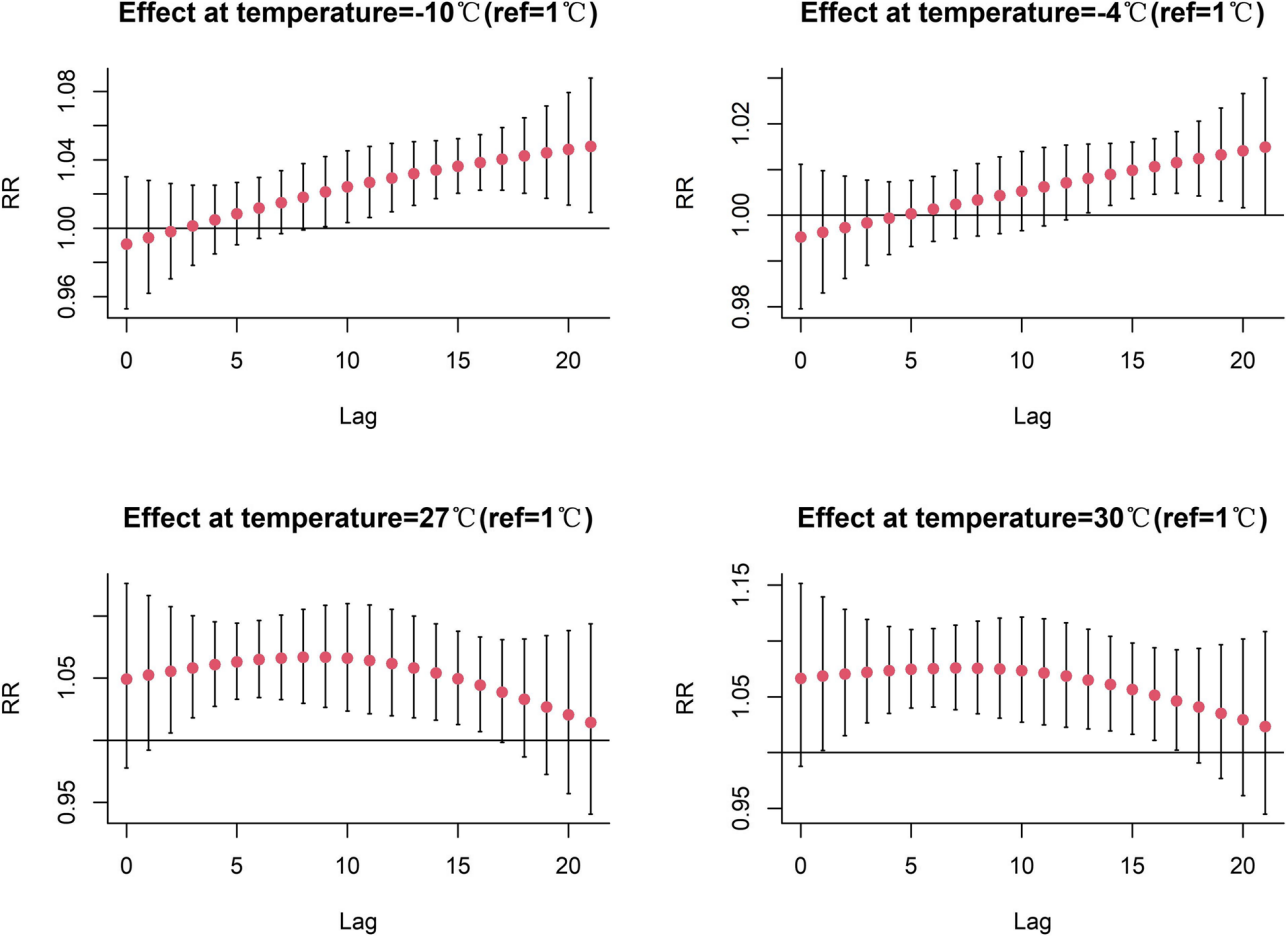
The lagged effect at specific temperatures on ED visits for respiratory diseases in Beijing

Figure 2 shows the lag effect at different specific temperatures (see Supplementary Table S1 for the corresponding table). In extremely cold weather, the RR values from lag 8 days to lag 21 days were greater than 1. Moreover, the RR for both extremely cold weather and moderately cold weather peaked at lag 21 days, 1.048 [95% confidence interval (CI): 1.009,1.088] and 1.015 (95% CI: 1.000,1.030), respectively. At a 7-day lag in extremely hot weather, the RR peaked at 1.076 (95% CI: 1.038, 1.114), and at an 8-day lag in moderately hot weather, it peaked at 1.067 (95% CI: 1.030, 1.105).

Figure S6 demonstrates the trend of the risk of respiratory diseases with different temperatures for a specific number of lag days. At a lag of 3 days, respiratory diseases were at high risk at hot temperatures, i.e., hot temperatures showed mainly short lag effects. At a lag of 21 days, respiratory diseases were at high risk at cold temperatures, i.e., cold temperatures mainly showed a long lag effect.

**Fig. 3:**
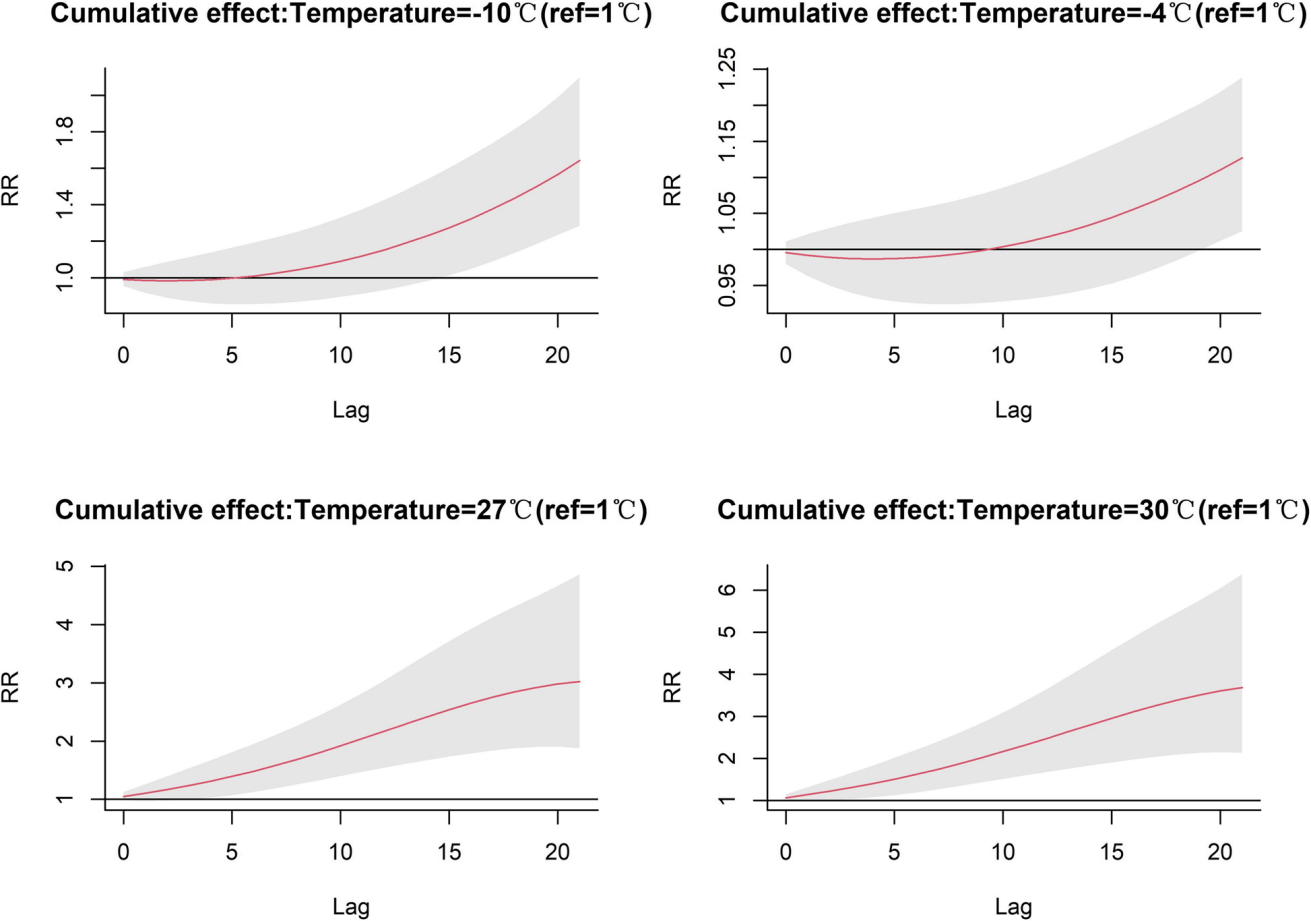
The estimated cumulative effect of specific temperatures on respiratory diseases incidence in Beijing

Figure 3 and Table S2 show the cumulative relative risk of hot and cold effects at different lag days. The cumulative risk of morbidity was higher in extremely cold weather than in moderately cold weather, peaking at 1.641 (95% CI: 1.284, 2.098) on days 0–21. The moderate cold started with a cumulative RR greater than 1 at the 0–10 days cumulative lag and peaked at 1.127 (95% CI: 1.025, 1.239) at 0–21 days. In extremely hot weather, the cumulative risk of morbidity was higher than in moderately hot conditions. The cumulative RR for extremely hot weather was greater than 1 at the beginning of the cumulative lag period of 0–2 days, and reached a maximum at 0–21 days for both extremely and moderately hot weather, 3.690 (95% CI: 2.133, 6.382) and 3.023 (95% CI: 1.878, 4.866), respectively. The cumulative lagged effects of the cold and hot effects generally increased gradually with increasing cumulative lag days, and the hot effect was more pronounced than the cold effect, and both reached a maximum cumulative incidence risk at lags of 0–21 days.

### Subgroup analysis

**Fig. 4:**
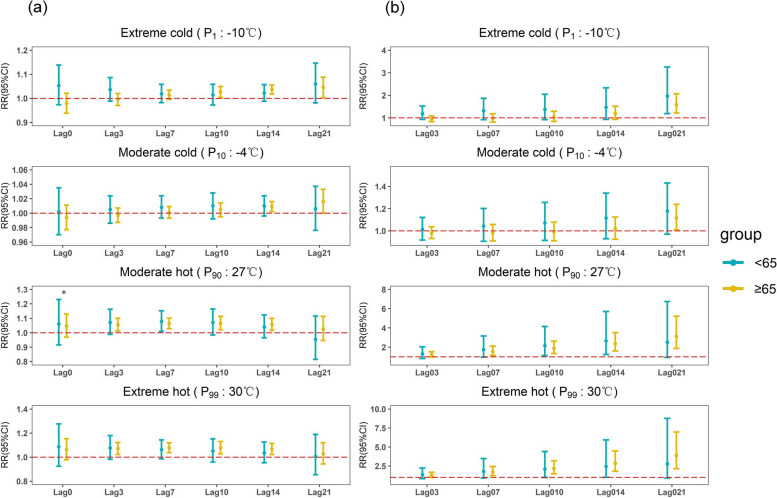
The effect of ambient temperature on respiratory ED visits by age. **a** Single-day lagged effect of average daily temperature on respiratory ED visits. **b** Cumulative lagged effect of average daily temperature on respiratory ED visits. *p < 0.05)

**Fig. 5:**
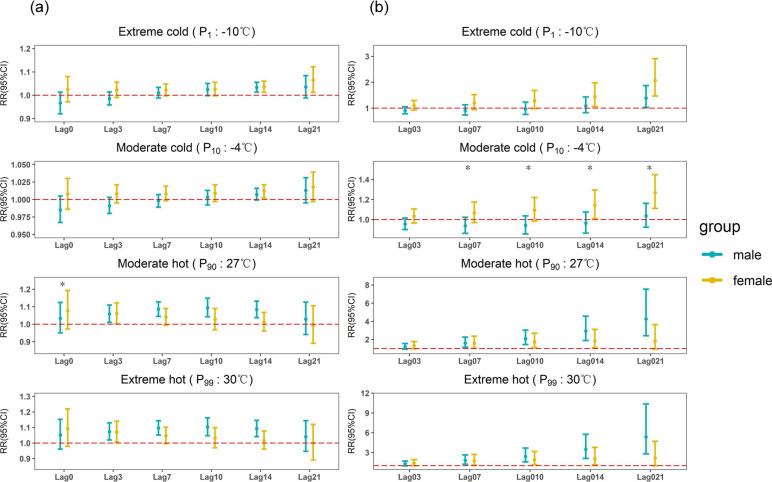
The effect of ambient temperature on respiratory ED visits by gender. (**a** Single-day lagged effect of average daily temperature on respiratory ED visits. **b** Cumulative lagged effect of average daily temperature on respiratory ED visits. *p < 0.05)

Subgroup analyses of people of different ages (< 65 years, ≥ 65 years) and genders (male, female) in Figs. 4 and 5 showed that hot temperature showed mainly a short lag effect, with a risk of morbidity at a lag of 3 days and not exceeding a lag of 14 days; cold temperature showed mainly a long lag effect, with a risk of morbidity observed at a minimum lag of 14 days and even at a lag of 21 days (Supplementary Table S3). All categories of the population were more sensitive to hot weather compared to cold weather, especially extremely hot weather. In particular, the cumulative risk of respiratory diseases was more affected by extremely hot weather at a lag of 0–21 days in the population aged 65 years or older as well as in the male population (Supplementary Table S4, Figure S7). But there was no statistically significant difference between the two age groups or between men and women (*p* = 0.835 and *p* = 0.594). In hot weather, the risk was greater for men than for women, 1.096 (95% CI: 1.051, 1.143), while cold weather had a longer-lasting effect on women than on men, with a relative risk of 1.065 (95% CI: 1.012, 1.122). But the difference between men and women is not statistically significant (*p* = 0.171 and *p* = 0.413). Women are more sensitive to moderate cold than men. The relative risk of emergency visits for female respiratory diseases reached 1.142 (95% CI: 1.008, 1.294) when there was a cumulative lag of 0–14 days, and 1.268 (95% CI: 1.110, 1.447) when there was a cumulative lag of 0–21 days, with a statistically significant difference between males and females (*p* = 0.043 and *p* = 0.023).

### Meta-analysis

**Fig. 6:**
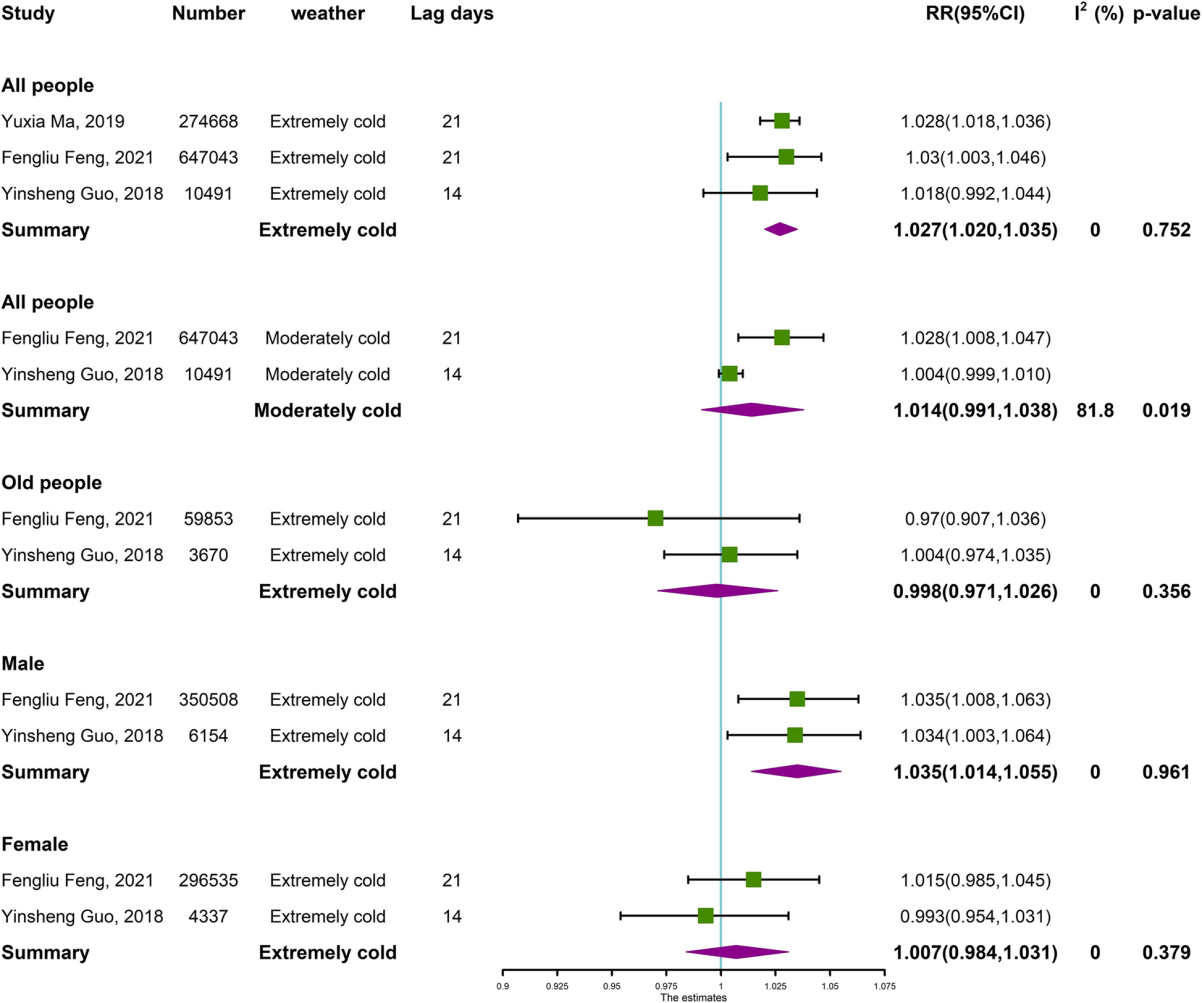
Meta-analysis of cold effects on the risk of respiratory ED visits in China

**Fig. 7:**
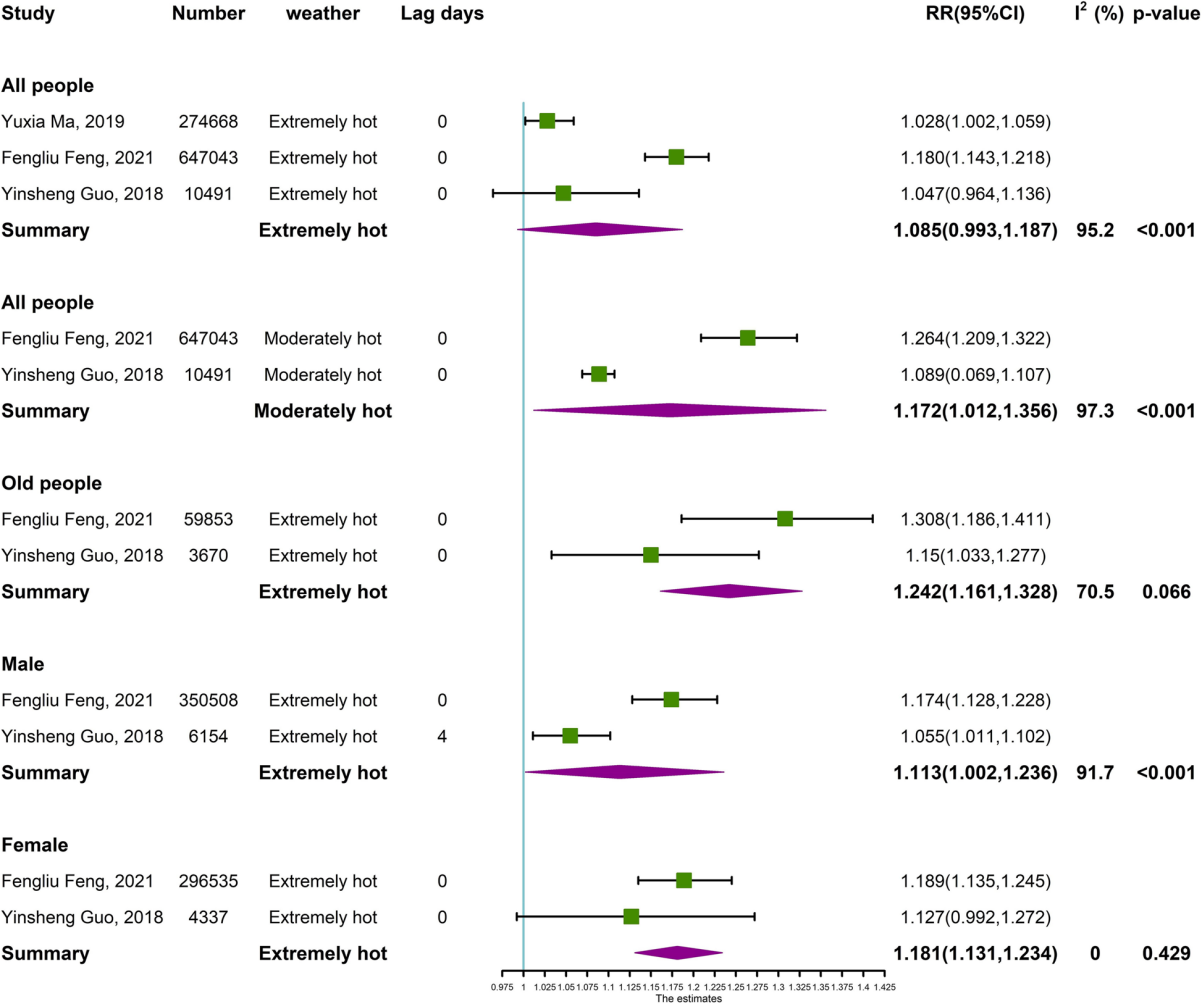
Meta-analysis of hot effects on the risk of respiratory ED visits in China

A total of three eligible papers from Lanzhou [35], Shenzhen [36], and Beijing [22], China, representing northwest, south, and north China, respectively, were finally included. Meta-basic information tables are shown in Supplementary Table S5, S6. The effects of cold and hot conditions on the risk of respiratory diseases in various populations are discussed in Figs. 6 and 7, respectively. The overall effect of cold weather in the three papers (Fig. 6) showed that the risk of respiratory diseases was greater and statistically significant at 1.027 (95% CI: 1.020, 1.035) for long lags of extreme cold weather compared to moderately cold weather. Among the effects of cold weather on various subgroups of the population, the effect was more pronounced in men, at 1.035 (95% CI: 1.014, 1.052). However, in the present study, the effect of cold weather was more pronounced in women at 1.065 (95% CI: 1.012, 1.122). As shown by the overall effect under hot weather in the three papers (Fig. 7), the risk of respiratory diseases was higher under a short lag of moderately hot weather at 1.172 (95%CI: 1.012, 1.356). The risk of respiratory diseases was higher in the elderly and in both men and women with a short lag of extreme hot, with a higher risk in women than in men, 1.181 (95% CI: 1.131, 1.234). However, in the present study, the effect of hot was greater for men, at 1.071 (95% CI: 1.007, 1.140). Overall, the hot had a greater impact on the risk of respiratory diseases than the cold.

### Sensitivity analysis

To test the robustness of the model in this study. First, the degrees of freedom of the natural cubic spline function in the crossover base of the DLNM model were adjusted for the exposure dimension and the lag dimension. The values in the preceding analysis are 4 and 3. When increasing the degrees of freedom in the two dimensions from 2 to 6, the impact values do not vary appreciably (Supplementary Figure S8). Second, the interaction of pm2.5 with daily average temperature, the interaction of SO2 with daily average temperature, and the combined effect of the above two interactions did not have a significant effect on the results (Supplementary Figure S9). Finally, after setting the maximum lag days to 10 days, it was found that the cumulative lag effect at different temperatures did not change much from the maximum lag days of 21 days (Supplementary Table S7, Figure S10). The sensitivity analysis findings show that the parameters of the DLNM model constructed in this study were reasonably chosen, and the findings of model fitting were stable.

## Discussion

In this study, the association between the daily mean temperature and ED visits for respiratory diseases was analyzed in Beijing during 2017–2018 and a meta-analysis of the literature on similar study conditions was performed in China. In general, hot weather exhibited a stronger effect on respiratory admissions. On the other hand, the effect of low temperatures on these admissions seemed to prevail for a longer time than did exposure to high temperatures. This observation is consistent with the findings of earlier investigations [37–39]. Extreme temperatures seemed to affect respiratory ED visits most of all studied temperature ranges.

Substantial research has already proven a significant connection between ambient temperature and respiratory disease mortality. A study from Mianyang City found that the exposure–response curve between daily mean temperature and mortality from respiratory diseases showed a "V"-shaped nonlinear feature, with both cold and hot temperatures increasing the risk of mortality from respiratory diseases [40], and its findings were similar to those of several other studies [13, 25, 41, 42]. However, there are limitations in the investigation of the relationship between ambient temperature and mortality. There may be discrepancies between the actual time of death and reported events, which has an impact on the accuracy of the results. Also, mortality rates usually reflect the health status of those with severe conditions and are not suitable for studying the direct impact of temperature on the general population [37]. Emergency visits may therefore be a more meaningful indicator for capturing the exact incidence of respiratory disease [43]. Numerous national and international research has demonstrated the influence of hot and cold ambient temperatures on emergency visits, outpatient visits, and hospital admission rates for respiratory diseases [24, 28, 44, 45]. Consistent with the above studies, data from the present study showed a significant positive association between ambient hot weather and cold weather for both respiratory diseases ED visits, with the effect of hot being more pronounced, and this result was confirmed in the meta-analysis. However, the mechanisms underlying this relationship have not been fully elucidated, and the following mechanisms may explain the effect of temperature on the respiratory system. Most previous laboratory and clinical studies have demonstrated that ambient temperature can directly affect the risk of respiratory diseases not only by stimulating respiratory mucosal vasoconstriction and suppressing the immune response [46, 47] but also by directly affecting bacterial pathogen activity and patient outdoor activity time and thus indirectly induce respiratory events [48, 49]. In addition to this, many epidemiological studies showed significant direct and indirect adverse effects of air pollutants on respiratory mortality, such as PM2.5 and SO2 [50, 51], and the interaction between temperature and pollutants may affect health outcomes. For example, PM2.5 composition varies significantly from source to source [52] and has different effects on pathogenic microorganisms [53] through complex interactions and transformations of temperature, humidity, and other factors; also, low-temperature conditions can cause temperature inversion, which impede the diffusion of pollutants [54]. All these can lead to increased incidence of respiratory diseases. Besides environmental factors, a number of existing respiratory diseases may influence vulnerability to ambient temperature and increase the risk of respiratory ED visits. Some studies have confirmed that either high or low temperatures are triggers for asthma attacks [55], and lower temperatures are associated with an increased number of COPD exacerbations [56].

However, the results of this study are not consistent with some previous studies. A study located in Shiraz, Iran, found that cold temperatures increased respiratory-related deaths, but no effect of hot temperatures on respiratory mortality was observed [57]. The reason for the existence of this situation may be that Shiraz is a city with a mild climate and therefore, people in this city are more adapted to warm weather compared to cold weather. In Dongguan, China, although a large proportion (8.4%) of respiratory outpatient visits could be attributed to ambient cold or hot temperatures during the study period, the majority of the burden of respiratory diseases were caused by moderate hot exposure (7.5%) [47]. This may be because Dongguan is located in a subtropical region where the definition of ambient cold temperature is higher than that of our study, and people often choose not to go out and cool themselves indoors through air conditioning during extremely hot temperatures. In addition, the result that cold temperatures had a more pronounced impact on the increase in clinical visits related to respiratory diseases compared to high temperatures, especially extremely cold temperatures were observed in two poor counties in Ningxia Hui Autonomous Region [58]. This may be because the climate of Ningxia is characterized by long, cold winters and cool, less intense summers, while poor counties have low heating and air conditioning penetration rates and are therefore more sensitive to long, cold winters.

The lag effect analysis results in this study show that the cold weather impact is greatest at lags of 21 days and 0–21 days, while the hot weather effect is greatest at lags of 7 days and 0–21 days. This indicates that cold weather appears to have a longer impact on respiratory admissions than exposure to hot weather, but the hot effect is more pronounced and has a greater effect than the cold effect. A possible reason for this outcome is that temperature, in addition to directly influencing respiratory morbidity by triggering vascular changes, releasing inflammatory mediators, and decreasing the effectiveness of the immune response [59, 60], can also indirectly induce respiratory events, such as influencing bacterial or viral activity [61], which can affect respiratory infections due to exposure to pathogens and develop into pneumonia over time. This result of the present study is similar to a study in Tibet, which found that hot temperatures had a greater effect on morbidity than cold temperatures and that the acute hot effect at lag 0 was associated with an increase in respiratory diseases, RR = 1.119 (95% CI: 1.010–1.240) [23]. Not coincidentally, three studies from Lanzhou, China, the Klang Valley region of Malaysia, and Chiang Mai, Thailand, also discovered that the effects of low temperatures on respiratory admissions seem to last longer compared to exposure to high temperatures [37–39].

Our study confirms that older people over 65 years old are more susceptible to ambient temperature, especially hot temperatures. This is consistent with the results of the meta-analysis and several other studies [26, 38, 41, 62]. There are two possible explanations for this result. First, the poorer immunity of the elderly and the fact that the regulation of body temperature involves several organ systems and that the thermoregulatory capacity decreases with the aging process make the elderly more vulnerable to environmental hyperthermia [63]. And the high prevalence of chronic diseases in the elderly and physiological impairments may have diminished their ability to cope with hot temperatures [64]. Across gender subgroups, this study observed that men are more vulnerable to hot weather than women [12], which might be explained by the fact that men are more prone to participate in outdoor activities than women and that men may have a less healthy lifestyle, such as men who consume excessive alcohol or are more exposed to tobacco [65]. This study also observed that women were affected by cold weather for a longer duration than men, which could relate to possible differences in anthropometry favoring body cooling, health or behavior between genders [40]. However, unlike the findings of this study's gender subgroup, the meta-analysis found that, while both cold and hot weather were significant for men, women were more vulnerable to the impacts of hot weather. Therefore, further exploration is needed regarding the effect of ambient temperature in each region on the development of respiratory diseases in different gender groups.

The advantages of our study are obvious. To our knowledge, this is the first study to combine the DLNM model with meta-analysis to analyze the relationship between the different levels of ambient temperature and respiratory ED visits. In addition, in the context of socio-economic and ecological changes, the relationship between ambient temperature and ED visits for respiratory diseases was explored. Various temperature levels, age-specific, as well as gender-specific effects were also provided, which suggested sufficient information for decision support and targeted prevention and control. But it should be acknowledged that there are also certain limitations to this study. First, this is an ecological study and no personal exposure data were available, so a degree of ecological fallacy is inevitable. Second, the time series of this study was short (two years), and although some compensation was made in the meta-analysis, this study did not collect data for a longer time series to control for the effect of abnormal seasons on effect estimates. In addition, respiratory diseases are also affected by indoor ambient temperature and pollutants, however, due to the difficulty of collecting data on the indoor temperature where individuals are located, this study was unable to control for the potential corrective effect of indoor temperature, and therefore the results may be confounded.

## Conclusion

In conclusion, our study shows that either cold or hot ambient temperatures have a negative impact on the risk of ED visits for respiratory diseases. Hot weather had a more acute impact on the number of hospital admissions for respiratory diseases, whereas low temperatures appear to have a longer impact on respiratory admissions than exposure to high temperatures. Older adults are more sensitive to ambient temperature and are particularly vulnerable to extremely hot temperatures. Men are more susceptible to extremely hot weather than women, while women are affected by cold weather for longer durations than men. However, further investigation is needed regarding the effect of ambient temperature on the onset of respiratory diseases by gender. Our study suggests that with the future trend of global warming, there is an urgent need for relevant authorities in Beijing and similar climatic regions to introduce relevant protective and preventive measures at the practical and policy levels, which are important for improving global public health strategies.

### Supplementary Information

Supplementary Material 1.

## Data Availability

The data that support the findings of this study are available from the corresponding author, upon reasonable request.

## Abbreviations

ED: Emergency departments
GAM: Generalized additive model
DLNM: Distributed lagged non-linear model
CI: Confidence Interval
RR: Relative risk
NO_2_: Nitrogen dioxide
SO_2_: Sulfur dioxide
O_3_: Ozone
CO: Carbon monoxide
RH: Relative humidity
SD: Sunshine duration
DF: Degree of freedom
GCV: Generalized cross-validation
