# Supplementary material for: Lag effect of ambient temperature on respiratory emergency department visits in Beijing: a time series and pooled analysis
Source: BMC Public Health. 2024 May 21;24:1363. doi: 10.1186/s12889-024-18839-6 (PMC11106889; doi:10.1186/s12889-024-18839-6)
Supplement: Supplementary file 1 — Supplementary Material 1. [file 12889_2024_18839_MOESM1_ESM.pdf]

Lag effect of ambient temperature on respiratory emergency department visits in Beijing: A time series and pooled analysis

Xuan Li[1†], Yongming Zhang[2†], Zhenbiao Tian[3†], Jianping Wang[1], Jinhua Zhao[1], Yuanjun Lyu[4], Ying Ni[1], Yuming Guo[5], Zhuang Cui[1], Wenyi Zhang[6a], Changping Li[1a]

Affiliations of authors:

[1] Department of Epidemiology and Biostatistics, School of Public Health, Tianjin Medical University, Tianjin, China

[2] Department of Pulmonary and Critical Care Medicine, Center of Respiratory Medicine, China-Japan Friendship Hospital, National Clinical Research Center for Respiratory Diseases, Beijing, China.

[3] Beijing Red Cross Emergency Center, Beijing 100085, China.

[4] Department of Endocrinology, Tianjin Hospital, Tianjin, China

[5] Department of Epidemiology and Preventive Medicine, School of Public Health and Preventive Medicine, Monash University, Melbourne, Australia

[6] Chinese PLA Center for Disease Control and Prevention, Beijing, China

[†]These authors contributed equally to this study.

[a] **Corresponding author:**

Wenyi Zhang, Chinese PLA Center for Disease Control and Prevention, 20 Dong-Da Street, Fengtai District, Beijing 100071, People's Republic of China.

E-mail: zwy0419@126.com

Changping Li, Department of Epidemiology and Biostatistics, School of Public Health, Tianjin Medical University, Heping District, Tianjin,300070, P.R. China.

E-mail: ChangpingLi417@126.com

Supplemental Materials

Table of Contents	Page
**Fig. S1.** locations of major emergency stations and all monitoring stations	3
**Fig. S2.** Flow chart of Meta analysis.	4
**Fig. S3.** Time series distribution of daily respiratory ED visits and daily mean temperature in Beijing.	5
**Fig. S4.** Scatterplot of daily respiratory ED visits and daily mean temperature in Beijing.	6
**Fig. S5.** The graph of the sum of the absolute value of PACF and the df of time.	7
**Table S1** Relative risk of respiratory ED visits at specific ambient temperature.	8
**Fig S6.** Relationship between temperature and risk of respiratory diseases development at different lag days in Beijing.	9
**Table S2** Cumulative association and relative risk with different lag days of specific temperatures on respiratory ED visits.	10
**Table S3** The relative risk of respiratory diseases incidence risk on different subgroups at specific ambient temperature.	11
**Table S4** Cumulative relative risk of respiratory ED visits on different subgroups at specific ambient temperatures.	13
**Fig. S7**. Cumulative relative risk of respiratory ED visits on different subgroups at specific ambient temperatures.	15
**Table S5** Basic information of literature in Meta-analysis of cold effect	16
**Table S6** Basic information of literature in Meta-analysis of hot effect	17
**Fig. S8.** The sensitivity analysis of effect of daily mean temperature on respiratory ED visits with different related parameters in Beijing (Cumulative RR for lags 0-21 days at moderately cold temperatures (-4°C) for different lags or exposure dimensions).	18
**Fig. S9.** Sensitivity analysis of the effect of daily mean air temperature on respiratory ED visit rates in Beijing (the lag effect after correcting for the interaction of different air pollution factors interacting with daily mean temperature).	19
**Table S7** Cumulative relative risks of respiratory ED visits for specific temperatures with different lag days at a maximum lag of 10 days.	20
**Fig. S10** Cumulative relative risks of respiratory ED visits for specific temperatures with different lag days at a maximum lag of 10 days.	21

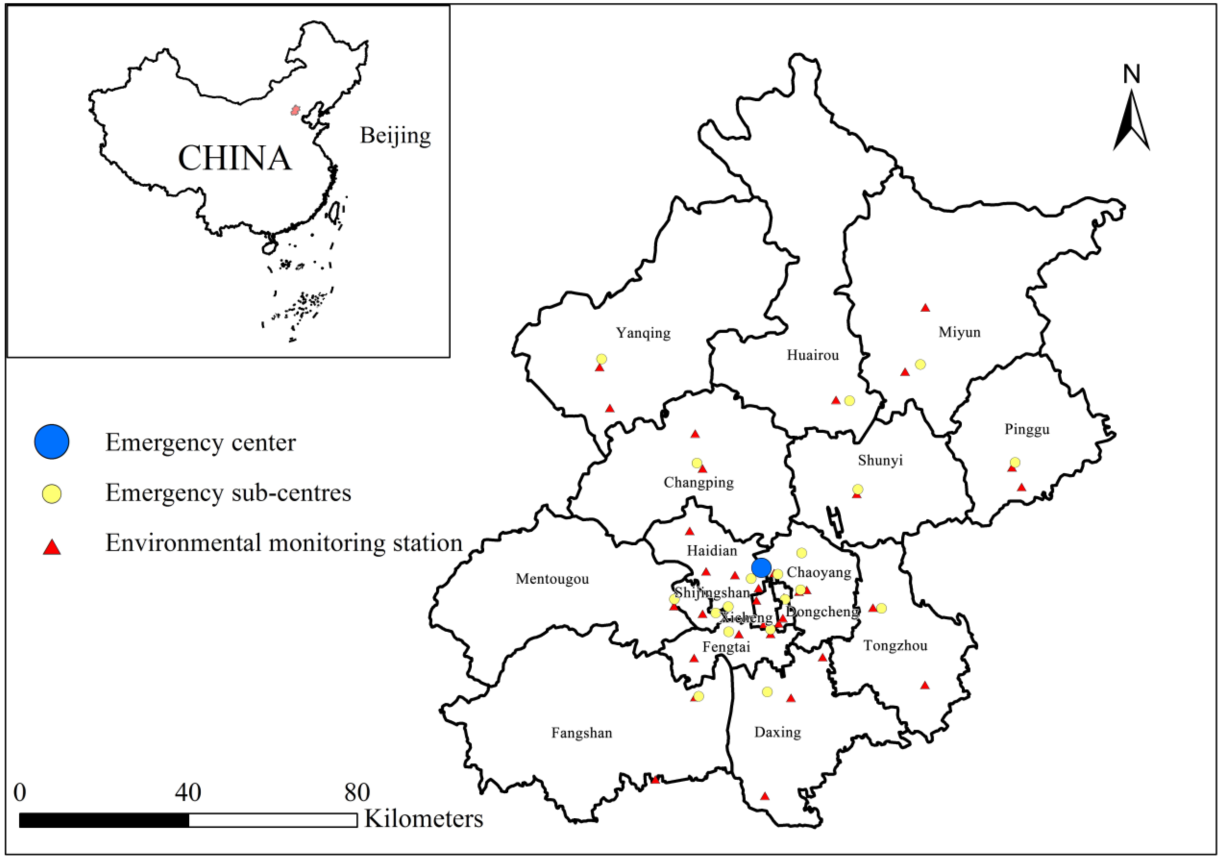

Fig. S1. Locations of major emergency stations and all monitoring stations

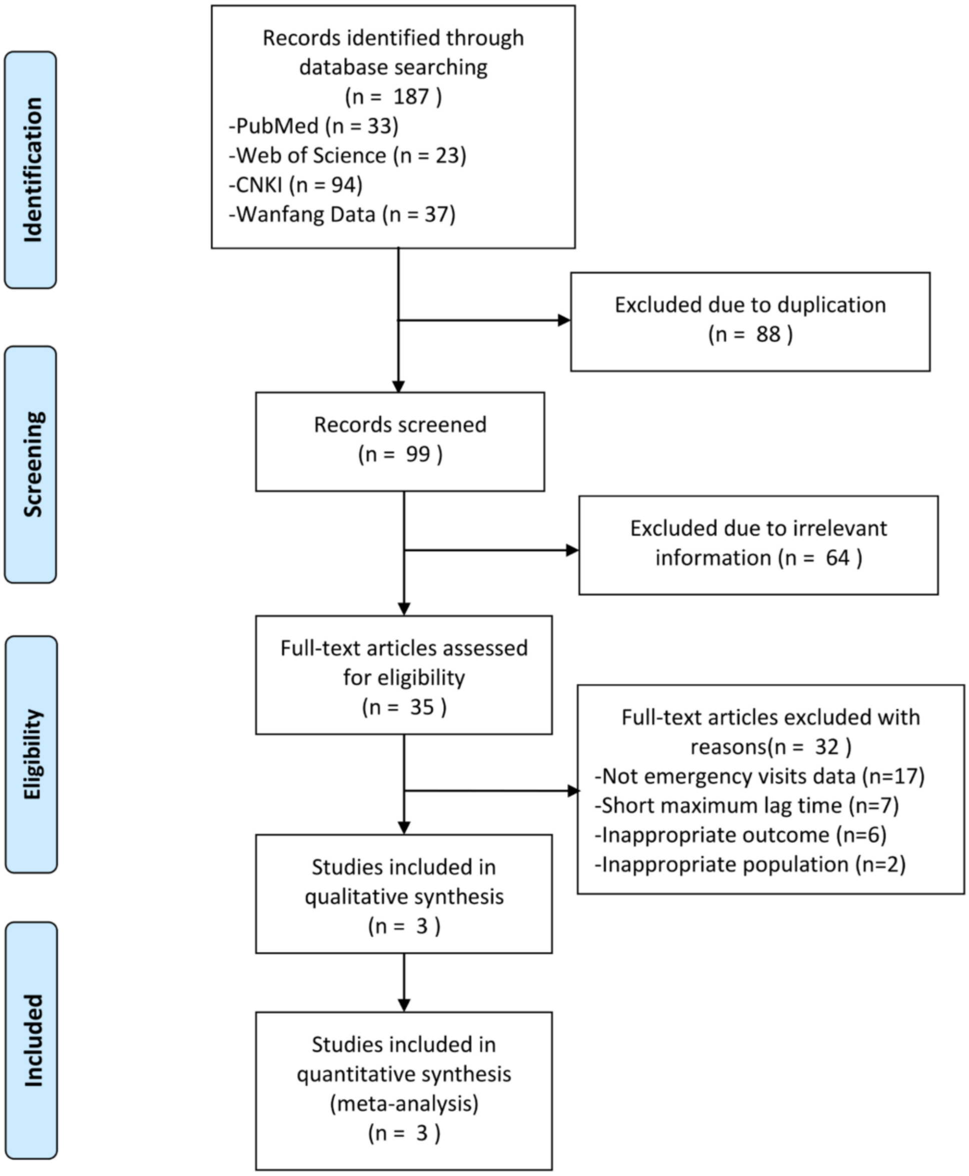

Fig. S2. Flow chart of Meta analysis.

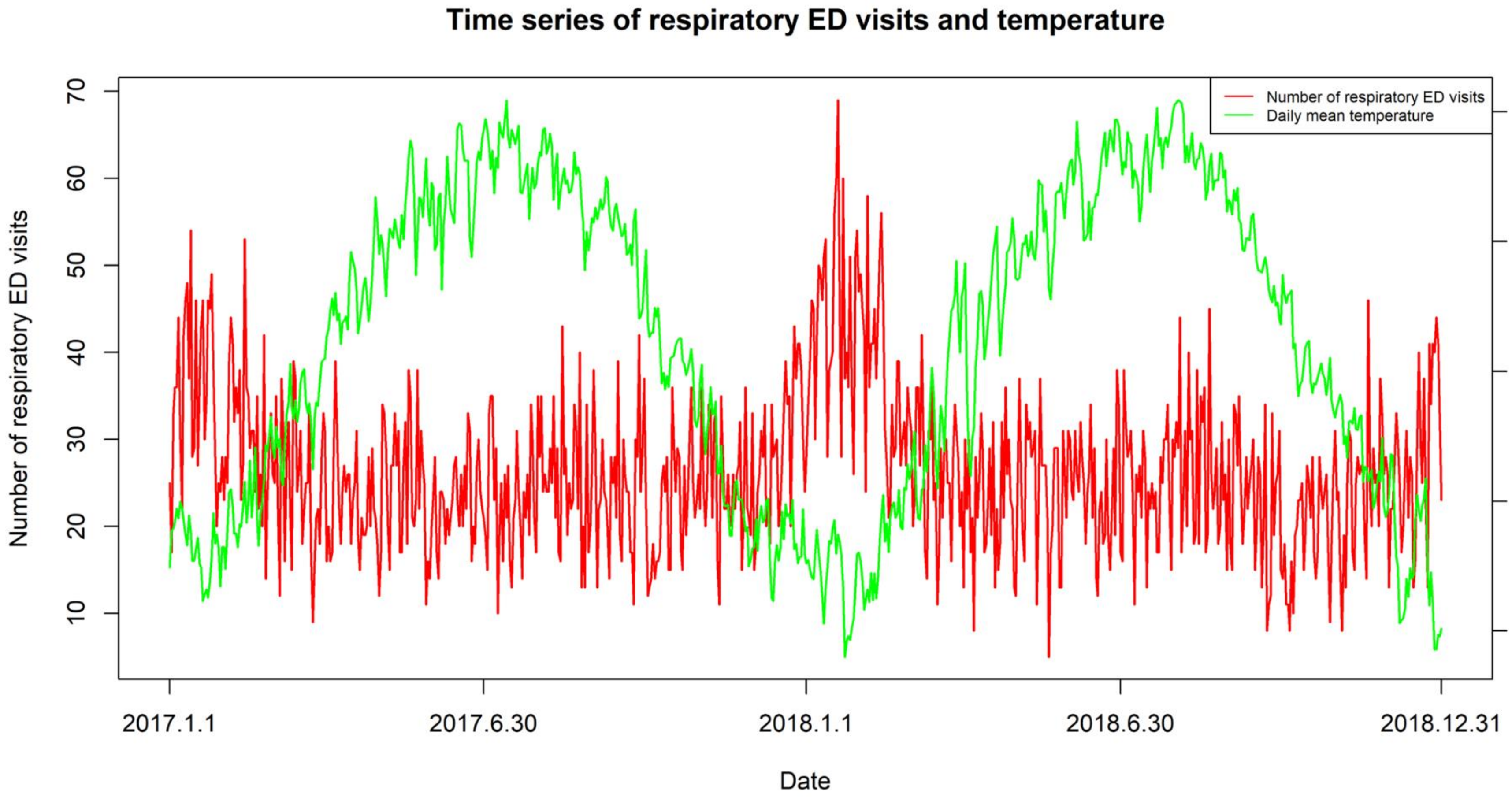

Fig. S3. Time series distribution of daily respiratory ED visits and daily mean temperature in Beijing.

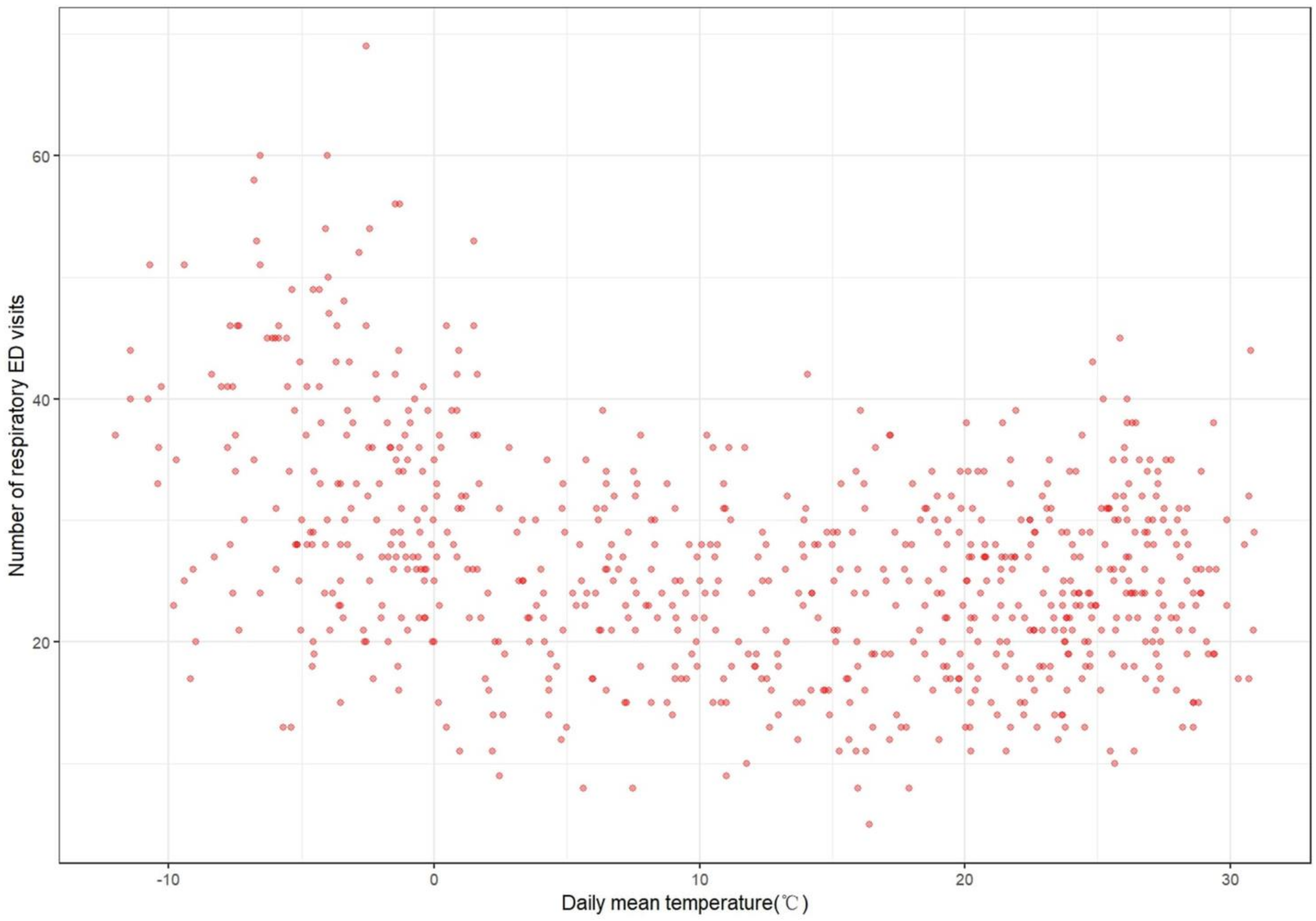

Fig. S4. Scatterplot of daily respiratory ED visits and daily mean temperature in Beijing.

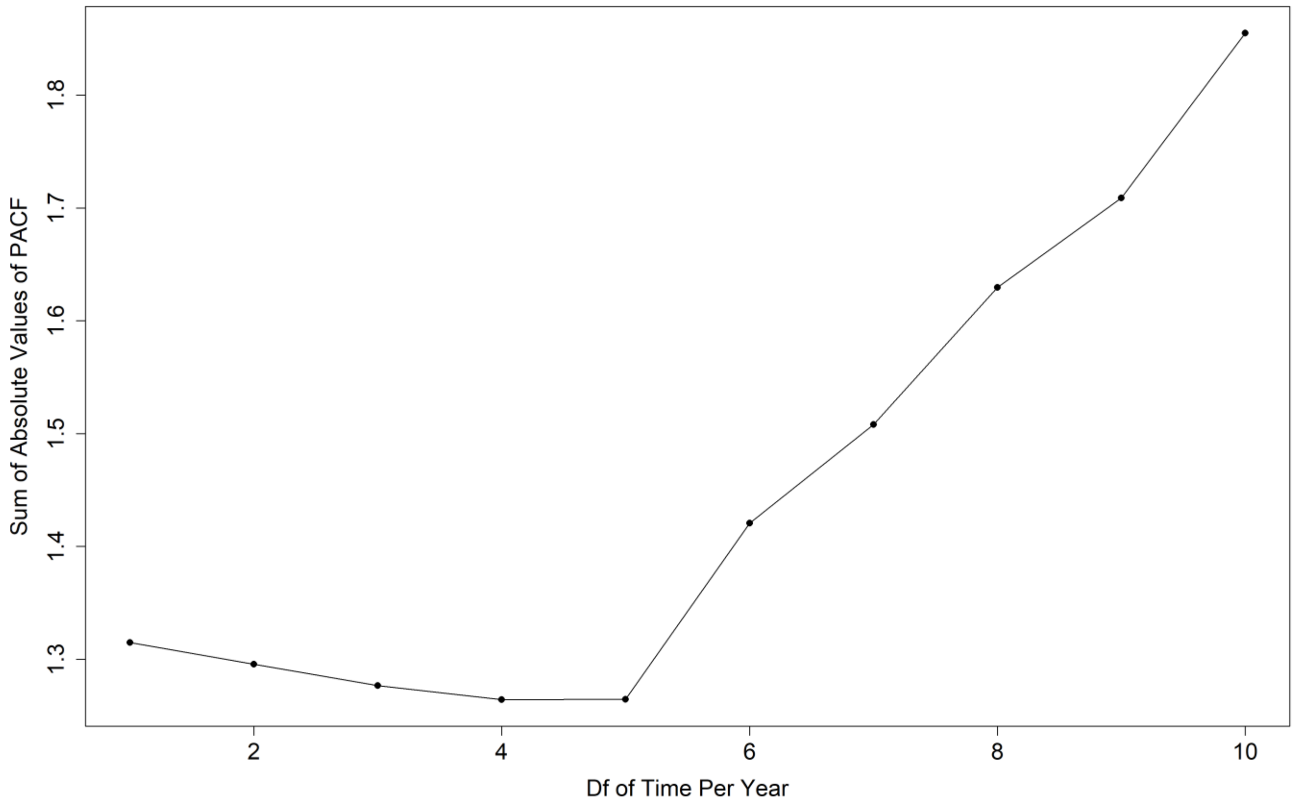

Fig. S5. The graph of the sum of the absolute value of PACF and the df of time.

Table S1

Relative risk of respiratory ED visits at specific ambient temperature.

	Extreme cold P_1:-10°C	Moderate cold P_{10}:-4°C	Moderate hot P_{90}:27°C	Extreme hot P_{99}:30°C
Lag 0	0.991 (0.953,1.030)	0.995 (0.980,1.011)	1.049 (0.978,1.126)	1.067 (0.988,1.151)
Lag 3	1.001 (0.978,1.025)	0.998 (0.989,1.008)	**1.058*** **(1.018,1.100)**	**1.072*** **(1.027,1.119)**
Lag 7	1.015 (0.997,1.034)	1.002 (0.995,1.010)	**1.066*** **(1.033,1.101)**	**1.076*** **(1.038,1.114)**
Lag 10	**1.024*** **(1.003,1.045)**	1.005 (0.997,1.014)	**1.066*** **(1.024,1.110)**	**1.073*** **(1.027,1.121)**
Lag 14	**1.034*** **(1.017,1.051)**	**1.009*** **(1.002,1.016)**	**1.054*** **(1.016,1.094)**	**1.061*** **(1.019,1.104)**
Lag 21	**1.048*** **(1.009,1.088)**	**1.015*** **(1.000,1.030)**	1.014 (0.941,1.094)	1.023 (0.945,1.108)

*p < 0.05 was considered statistically significant and is shown in bold type.

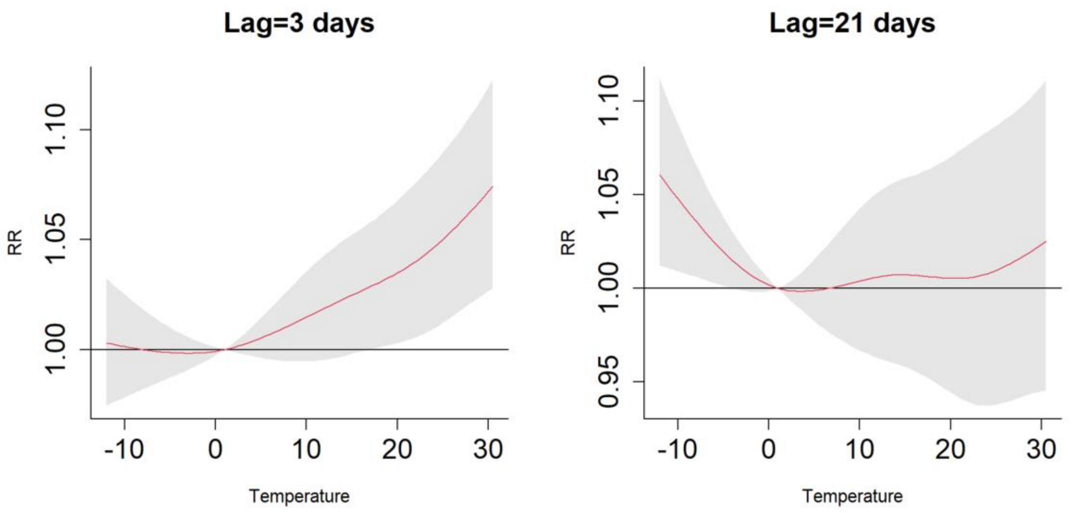

Fig S6. Relationship between temperature and risk of respiratory diseases development at different lag days in Beijing.

Table S2

Cumulative association and relative risk with different lag days of specific temperatures on respiratory ED visits.

	Extreme cold P_1:-10℃	Moderate cold P_{10}:-4℃	Moderate hot P_{90}:27℃	Extreme hot P_{99}:30℃
Lag0-3	0.984 (0.871,1.112)	0.987 (0.940,1.037)	1.234 (0.996,1.528)	**1.307*** **(1.034,1.652)**
Lag0-7	1.024 (0.860,1.220)	0.991 (0.924,1.062)	**1.580*** **(1.187,2.104)**	**1.744*** **(1.266,2.403)**
Lag 0-10	1.090 (0.895,1.329)	1.003 (0.928,1.086)	**1.917*** **(1.402,2.623)**	**2.164*** **(1.515,3.090)**
Lag0-14	1.230 (0.982,1.540)	1.034 (0.945,1.131)	**2.417*** **(1.673,3.492)**	**2.798*** **(1.836,4.265)**
Lag0-21	**1.641*** **(1.284,2.098)**	**1.127*** **(1.025,1.239)**	**3.023*** **(1.878,4.866)**	**3.690*** **(2.133,6.382)**

*p < 0.05 was considered statistically significant and is shown in bold type.

Table S3

The relative risk of respiratory diseases incidence risk on different subgroups at specific ambient temperature.

		Extreme cold P_1:-10℃	Moderate cold P_{10}:-4℃	Moderate hot P_{90}:27℃	Extreme hot P_{99}:30℃
Age					
<65	Lag 0	1.053 (0.973,1.138)	1.002 (0.970,1.035)	1.061 (0.915,1.230)	1.086 (0.924,1.276)
	Lag 3	1.036 (0.988,1.086)	1.005 (0.986,1.024)	1.072 (0.988,1.162)	1.076 (0.982,1.179)
	Lag 7	1.019 (0.982,1.058)	1.008 (0.993,1.024)	**1.078*** **(1.009,1.152)**	1.062 (0.986,1.143)
	Lag 10	1.014 (0.972,1.058)	1.010 (0.992,1.028)	1.071 (0.984,1.165)	1.051 (0.959,1.152)
	Lag 14	1.022 (0.988,1.057)	1.010 (0.996,1.024)	1.040 (0.964,1.123)	1.035 (0.953,1.125)
	Lag 21	1.060 (0.981,1.146)	1.006 (0.976,1.037)	0.954 (0.815,1.116)	1.007 (0.853,1.189)
≥65	Lag 0	0.979 (0.938,1.021)	0.994 (0.977,1.011)	1.046 (0.969,1.129)	1.062 (0.978,1.153)
	Lag 3	0.995 (0.970,1.020)	0.997 (0.987,1.007)	**1.055*** **(1.012,1.100)**	**1.070*** **(1.022,1.121)**
	Lag 7	1.014 (0.995,1.034)	1.001 (0.993,1.009)	**1.064*** **(1.028,1.101)**	**1.078** **(1.038,1.119)**
	Lag 10	**1.026*** **(1.004,1.049)**	1.005 (0.995,1.014)	**1.065*** **(1.020,1.113**	**1.077*** **(1.028,1.129)**
	Lag 14	**1.037*** **(1.018,1.055)**	**1.009*** **(1.002,1.016)**	**1.057*** **(1.016,1.100)**	**1.066*** **(1.021,1.113)**
	Lag 21	**1.045*** **(1.003,1.088)**	**1.016*** **(1.000,1.033)**	1.026 (0.946,1.113)	1.027 (0.943,1.119)
Gender					
Male	Lag 0	0.966 (0.920,1.013)	0.985 (0.967,1.005)	1.033 (0.950,1.124)	1.051 (0.960,1.152)
	Lag 3	0.986 (0.958,1.014)	0.991 (0.980,1.003)	**1.058*** **(1.010,1.109)**	**1.073*** **(1.019,1.130)**
	Lag 7	1.010 (0.988,1.033)	0.998 (0.989,1.007)	**1.085*** **(1.044,1.127)**	**1.096*** **(1.051,1.143)**
	Lag 10	1.024 (0.998,1.05)	1.003 (0.992,1.013)	**1.094*** **(1.042,1.148)**	**1.103*** **(1.047,1.162)**
	Lag 14	**1.033*** **(1.013,1.055)**	1.007 (0.999,1.016)	**1.084*** **(1.037,1.132)**	**1.092*** **(1.041,1.145)**
	Lag 21	1.035 (0.988,1.084)	1.013 (0.995,1.031)	1.029 (0.941,1.126)	1.040 (0.946,1.144)

Female	Lag 0	1.024 (0.971,1.080)	1.008 (0.986,1.030)	1.076 (0.972,1.192)	1.092 (0.978,1.219)
	Lag 3	1.022 (0.990,1.056)	1.008 (0.995,1.021)	**1.060*** **(1.003,1.121)**	**1.071*** **(1.007,1.140)**
	Lag 7	1.022 (0.997,1.048)	1.008 (0.998,1.019)	1.040 (0.994,1.089)	1.047 (0.996,1.101)
	Lag 10	1.025 (0.997,1.055)	1.009 (0.997,1.021)	1.027 (0.968,1.089)	1.032 (0.969,1.099)
	Lag 14	**1.036*** **(1.012,1.060)**	**1.012*** **(1.002,1.021)**	1.012 (0.960,1.067)	1.016 (0.960,1.077)
	Lag 21	**1.065*** **(1.012,1.122)**	1.018 (0.997,1.039)	0.992 (0.890,1.105)	0.999 (0.891,1.119)

*p < 0.05 was considered statistically significant and is shown in bold type.

Table S4
Cumulative relative risk of respiratory ED visits on different subgroups at specific ambient temperatures.

		Extreme cold P1:-10℃	Moderate cold P10:-4℃	Moderate hot P90:27℃	Extreme hot P99:30℃
Age					
<65	lag0-3	1.188 (0.93,1.518)	1.014 (0.917,1.121)	1.293 (0.826,2.024)	1.365 (0.833,2.236)
	lag0-7	1.311 (0.922,1.864)	1.043 (0.905,1.201)	1.737 (0.954,3.161)	1.770 (0.900,3.481)
	lag0-10	1.372 (0.919,2.048)	1.072 (0.914,1.257)	**2.152*** **(1.119,4.14)**	2.076 (0.979,4.403)
	lag0-14	1.472 (0.931,2.33)	1.115 (0.929,1.339)	**2.651*** **(1.232,5.704)**	**2.440*** **(1.004,5.926)**
	lag0-21	**1.966*** **(1.188,3.253)**	1.178 (0.970,1.431)	2.506 (0.931,6.744)	2.783 (0.885,8.759)
≥65	lag0-3	0.948 (0.83,1.082)	0.982 (0.931,1.036)	1.218 (0.968,1.534)	**1.291*** **(1.004,1.661)**
	lag0-7	0.976 (0.807,1.179)	0.981 (0.909,1.057)	**1.544*** **(1.134,2.101)**	**1.727*** **(1.224,2.438)**
	lag0-10	1.043 (0.842,1.292)	0.991 (0.910,1.079)	**1.865*** **(1.331,2.613)**	**2.163*** **(1.475,3.174)**
	lag0-14	1.189 (0.933,1.516)	1.02 (0.925,1.124)	**2.362*** **(1.590,3.51)**	**2.847*** **(1.810,4.48)**
	lag0-21	**1.586*** **(1.216,2.067)**	**1.118*** **(1.009,1.238)**	**3.116*** **(1.866,5.205)**	**3.863*** **(2.142,6.967)**
Gender					
male	lag0-3	0.906 (0.779,1.053)	0.954 (0.898,1.013)	1.196 (0.927,1.544)	1.274 (0.963,1.685)
	lag0-7	0.909 (0.733,1.128)	0.937 (0.86,1.021)	**1.603*** **(1.138,2.257)**	**1.788*** **(1.218,2.624)**
	lag0-10	0.963 (0.755,1.229)	0.940 (0.854,1.036)	**2.086*** **(1.432,3.036)**	**2.389*** **(1.558,3.663)**
	lag0-14	1.086 (0.825,1.431)	0.962 (0.862,1.074)	**2.935*** **(1.888,4.564)**	**3.47*** **(2.093,5.753)**
	lag0-21	**1.386*** **(1.028,1.869)**	1.035 (0.922,1.161)	**4.255*** **(2.401,7.543)**	**5.359*** **(2.776,10.346)**
female	lag0-3	1.095 (0.927,1.295)	1.032 (0.964,1.105)	1.303 (0.957,1.773)	1.368 (0.978,1.914)
	lag0-7	1.194 (0.939,1.519)	1.066 (0.968,1.174)	**1.568*** **(1.04,2.363)**	**1.700*** **(1.075,2.689)**
	lag0-10	1.282 (0.976,1.683)	1.095 (0.982,1.22)	**1.719*** **(1.099,2.687)**	**1.894*** **(1.139,3.148)**
	lag0-14	**1.449***	**1.142***	**1.841***	**2.063***

	**(1.062,1.977)**	**(1.008,1.294)**	**(1.09,3.109)**	**(1.131,3.763)**
lag0-21	**2.065***	**1.268***	1.843	2.149
	**(1.466,2.909)**	**(1.110,1.447)**	(0.937,3.626)	(0.985,4.688)

*$p < 0.05$ was considered statistically significant and is shown in bold type.

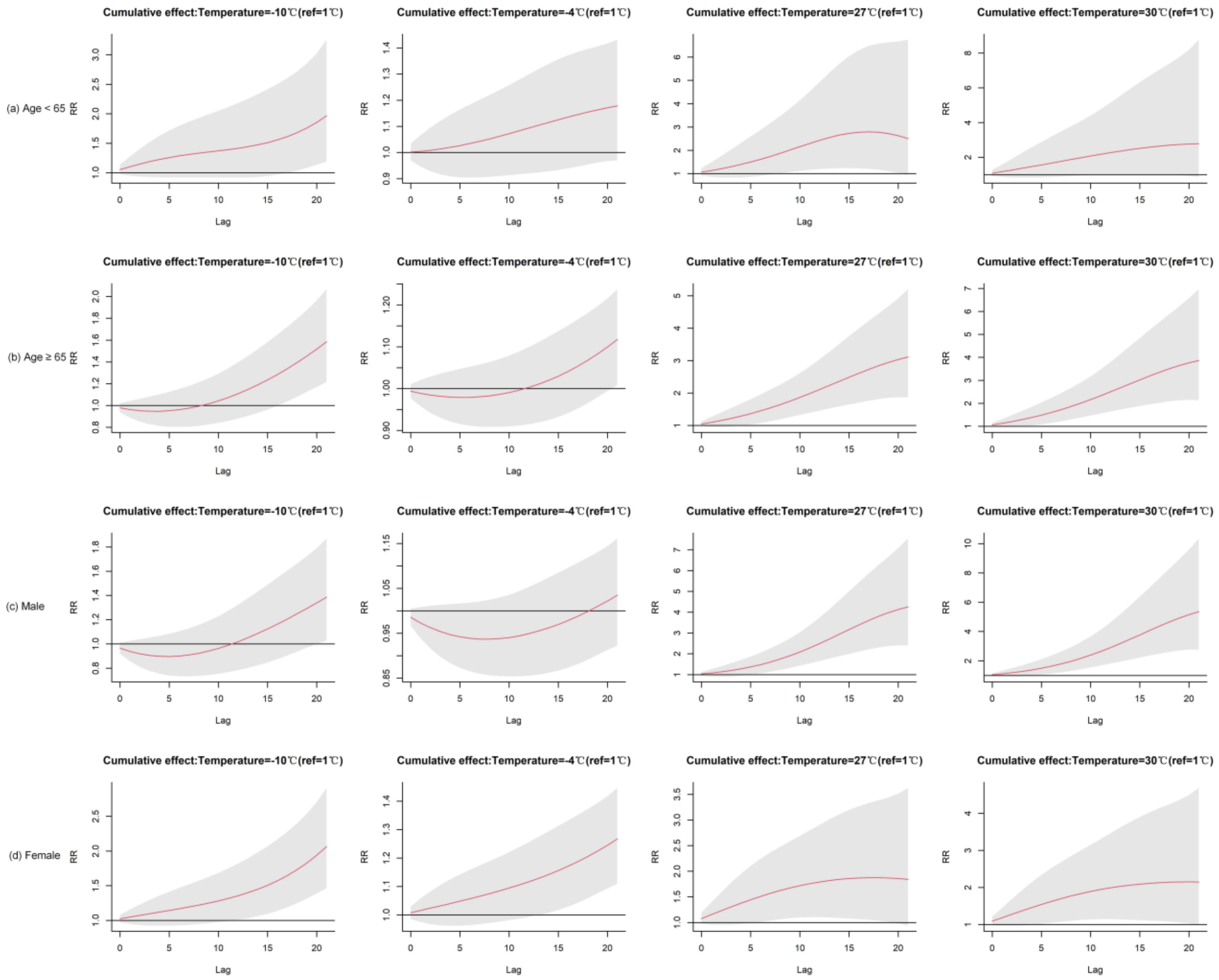

Fig. S7. Cumulative relative risk of respiratory ED visits on different subgroups at specific ambient temperatures.

Table S5

Basic information of literature in Meta-analysis of cold effect.

Literature	Study period	location	People	No. Events	Lag (Days)	Temperature percentile	Risk ratio and 95% confidence interval in cold days
Ma et al. 2019	2009-2012	Beijing	All	274668	21	P1	1.028(1.018,1.036)
Feng et al. 2021	2013-2017	Lanzhou	All	647043	21	P10	1.028(1.008,1.047)
			All	647043	21	P1	1.03(1.003,1.046)
			Old people(>60 years)	59853	21	P1	0.97(0.907,1.036)
			Male	350508	21	P1	1.035(1.008,1.063)
			Female	296535	21	P1	1.015(0.985,1.045)
Guo et al. 2018	2013-2017	Shenzhen	All	10491	14	P10	1.004(0.999,1.010)
			All	10491	14	P1	1.018 (0.992,1.044)
			Old people(≥65 years)	3670	14	P1	1.004 (0.974,1.035)
			Male	6154	14	P1	1.034 (1.003,1.064)
			Female	4337	14	P1	0.993(0.954,1.031)

Table S6

Basic information of literature in Meta-analysis of hot effect.

Literature	Study period	location	People	No. Events	Lag (Days)	Temperature percentile	Risk ratio and 95% confidence interval in hot days
Ma et al. 2019	2009-2012	Beijing	All	274668	0	P99	1.028(1.002,1.059)
Feng et al. 2021	2013-2017	Lanzhou	All	647043	0	P90	1.264(1.209,1.322)
			All	647043	0	P99	1.180(1.143,1.218)
			Old people(>60 years)	59853	0	P99	1.308(1.186,1.411)
			Male	350508	0	P99	1.174(1.128,1.228)
			Female	296535	0	P99	1.189(1.135,1.245)
Guo et al. 2018	2013-2017	Shenzhen	All	10491	0	P90	1.089(0.069,1.107)
			All	10491	0	P99	1.047(0.964,1.136)
			Old people(≥65 years)	3670	0	P99	1.150(1.033,1.277)
			Male	6154	4	P99	1.055(1.011,1.102)
			Female	4337	0	P99	1.127(0.992,1.272)

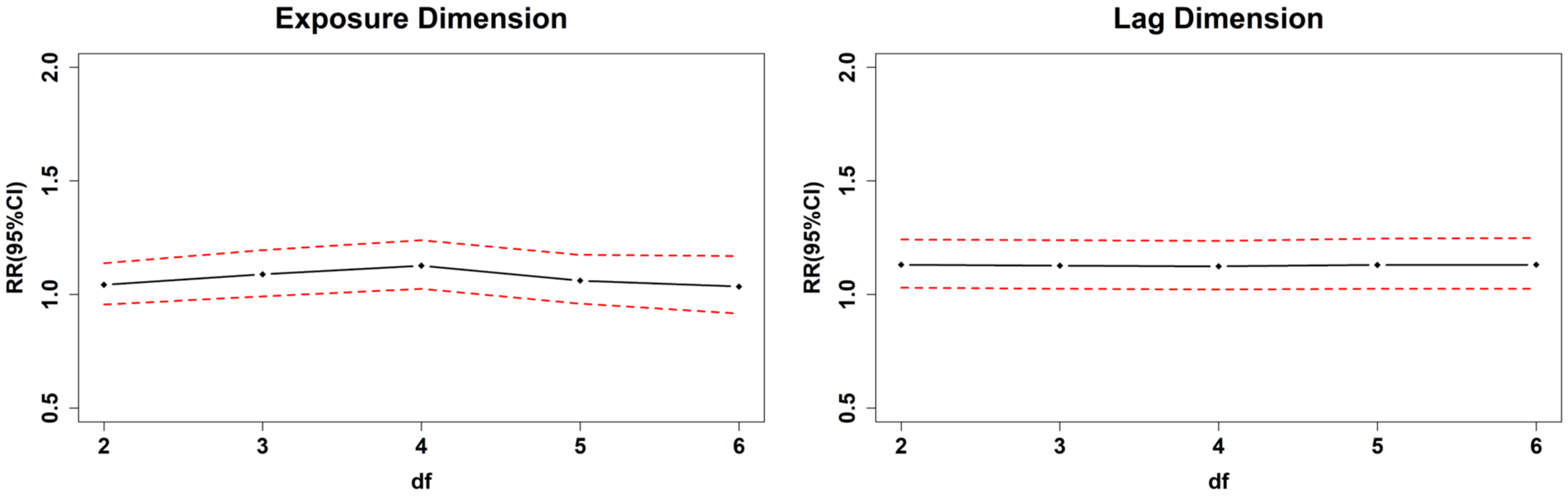

Fig. S8. The sensitivity analysis of effect of daily mean temperature on respiratory ED visits with different related parameters in Beijing (Cumulative RR for lags 0-21 days at moderately cold temperatures (-4°C) for different lags or exposure dimensions).

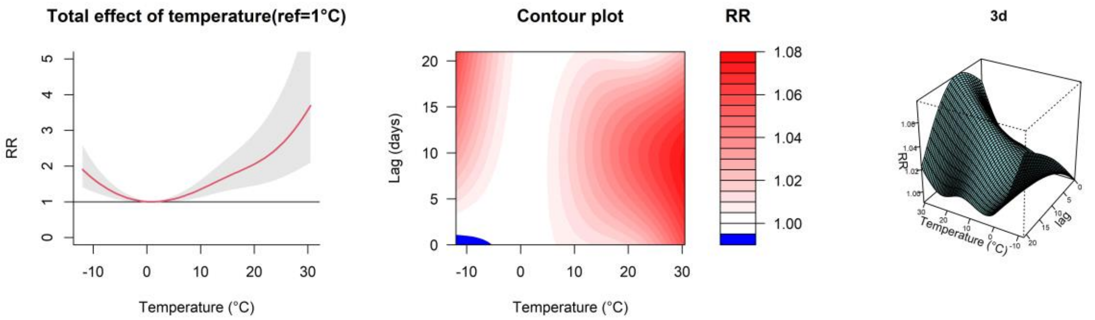

(a)The lag effect results including the interaction between $PM_{2.5}$ and daily mean temperature

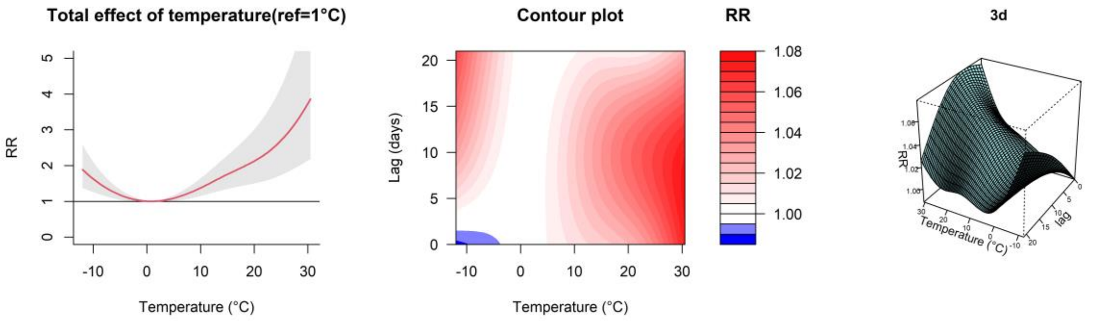

(b)The lag effect results including the interaction between SO_2 and daily mean temperature

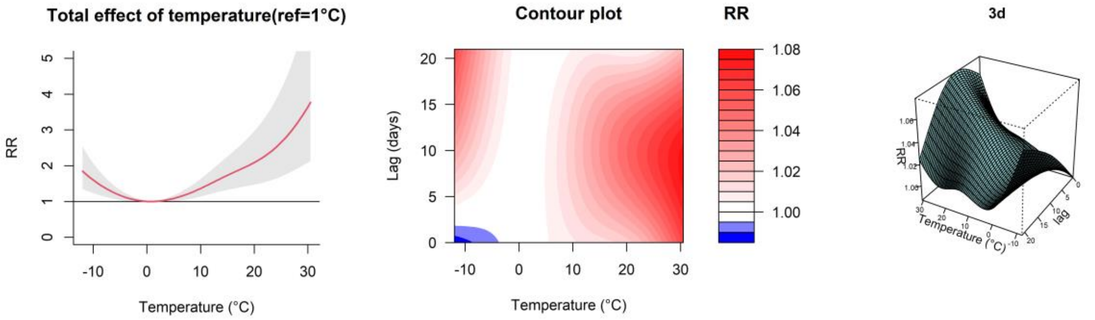

(c) Lag effect results including the interaction of $PM_{2.5}$ with daily mean temperature and the interaction of SO_2 with daily mean temperature

Fig. S9. Sensitivity analysis of the effect of daily mean air temperature on respiratory ED visit rates in Beijing (the lag effect after correcting for the interaction of different air pollution factors interacting with daily mean temperature).

Table S7 Cumulative relative risks of respiratory ED visits for specific temperatures with different lag days at a maximum lag of 10 days.

	Extreme cold P_1:-10℃	Moderate cold P_{10}:-4℃	Moderate hot P_{90}:27℃	Extreme hot P_{99}:30℃
Lag0-3	1.032 (0.896,1.187)	0.998 (0.943,1.056)	**1.351*** **(1.048,1.743)**	**1.412*** **(1.064,1.874)**
Lag0-7	**1.192*** **(1.015,1.399)**	1.050 (0.986,1.119)	**1.533*** **(1.149,2.044)**	**1.704*** **(1.234,2.355)**
Lag 0-10	**1.431*** **(1.181,1.735)**	**1.110*** **(1.033,1.193)**	**2.140*** **(1.537,2.979)**	**2.389*** **(1.631,3.501)**

*$p < 0.05$ was considered statistically significant and is shown in bold type.

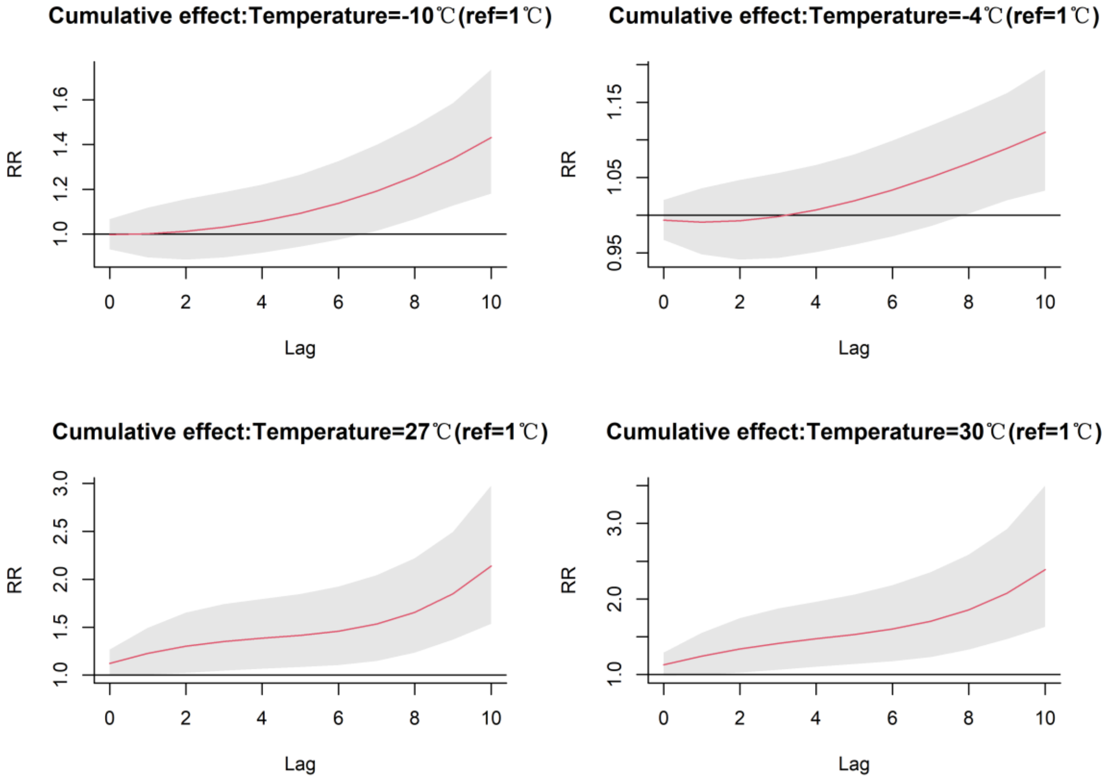

Fig. S10 Cumulative relative risks of respiratory ED visits for specific temperatures with different lag days at a maximum lag of 10 days.
